# Toward Modeling the Growth of Large Atmospheric Sulfuric Acid–Ammonia Clusters

**DOI:** 10.1021/acsomega.3c03521

**Published:** 2023-09-14

**Authors:** Morten Engsvang, Jakub Kubečka, Jonas Elm

**Affiliations:** † Department of Chemistry, Aarhus University, Langelandsgade 140, 8000 Aarhus C, Denmark; ‡ Department of Chemistry, iClimate, Aarhus University, Langelandsgade 140, 8000 Aarhus C, Denmark

## Abstract

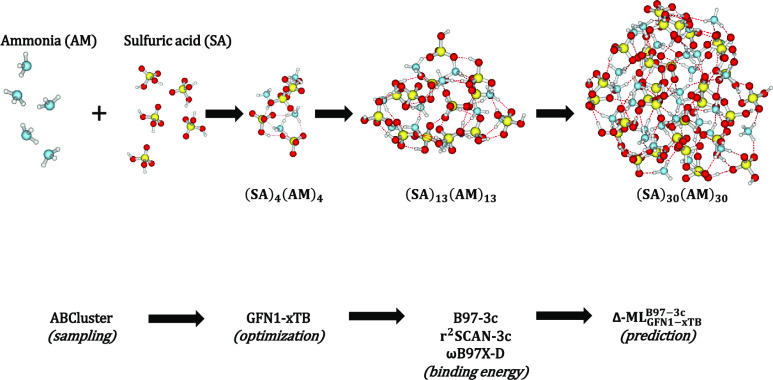

Studying large atmospheric molecular clusters is needed to understand the transition between clusters and aerosol particles. In this work, we studied the (SA)_*n*_(AM)_*n*_ clusters with *n* up to 30 and the (SA)_*m*_(AM)_*m*±2_ clusters, with *m* = 6–20. The cluster configurations are sampled using the ABCluster program, and the cluster geometries and thermochemical parameters are calculated using GFN1-xTB. The cluster binding energies are calculated using B97-3c. We find that the addition of sulfuric acid is preferred to the addition of ammonia. The addition free energies were found to have large uncertainties, which could potentially be attributed to errors in the applied level of theory. Based on DLPNO-CCSD(T_0_)/aug-cc-pVTZ benchmarks of the binding energies of the large (SA)_8-9_(AM)_10_ and (SA)_10_(AM)_10-11_ clusters, we find that ωB97X-D3BJ with a large basis set is required to yield accurate binding and addition energies. However, based on recalculations of the single-point energy at r^2^SCAN-3c and ωB97X-D3BJ/6-311++G(3df,3pd), we show that the single-point energy contribution is not the primary source of error. We hypothesize that a larger source of error might be present in the form of insufficient configurational sampling. Finally, we train Δ machine learning model on (SA)_*n*_(AM)_*n*_ clusters with *n* up to 5 and show that we can predict the binding energies of clusters up to sizes of (SA)_30_(AM)_30_ with a binding energy error below 0.6 %. This is an encouraging approach for accurately modeling the binding energies of large acid–base clusters in the future.

## 1 Introduction

The formation and growth of aerosols from gas-phase molecules is estimated to account for approximately half of all cloud condensation nuclei (CCN) present in the atmosphere.^1^ This means that understanding this process is crucial to reduce the still large uncertainty that the impact of aerosols has on the global climate,^2^ because CCN and aerosols, in general, can both negatively and positively affect the global energy budget. This can happen by the direct effect of absorbing light, as is the case with black carbon, the direct effect of scattering light, as is often the case for small salt aerosols, e.g., sulfuric acid–ammonia (SA–AM), and the indirect effect of acting as CCN, as is the case for aerosols of sufficient size and hygroscopicity.^3^

It has been shown that a clustering mechanism involving the formation of strong noncovalent bonds between acid and base molecules could be a potential route for the formation of new particles from gasses.^4^ One of the main acids thought to drive this cluster formation is sulfuric acid (SA).^5^ It is currently believed that the bases involved are likely to be atmospheric bases such as ammonia (AM),^6,7^ that is highly abundant in the atmosphere or the less abundant but much higher basicity amines such as methylamine (MA), dimethylamine (DMA), and trimethylamine (TMA).^8,9^ The global modeling study by Dunne et al.^10^ demonstrated that a large part of present-day new particle formation involves SA clustering with either AM or biogenic organic compounds.

Measurements of the initially formed clusters are difficult due to their small size. Techniques such as mass spectrometry can measure cluster compositions and gas concentrations.^11^ However, it is uncertain how accurate a reflection of the initially formed clusters they are due to them being relatively weakly bound compared to covalent bonds.^12^ Therefore, quantum chemical methods are necessary to map the gas–cluster–particle transition fully. Atmospheric cluster dynamics simulations of (SA)_*n*_(AM)_*n*_ clusters, with *n* up to 6, have shown that the clusters with 1:1 ratios between acid and base molecules are expected to be the most thermodynamically stable.^13−15^ In these clusters, it has also been shown that clusters with excess SA molecules are more stable than clusters with an excess of AM. The SA–AM cluster growth mechanism has been found to be an alternating addition of first an SA molecule and then an AM molecule.^13−15^ Furthermore, it has been demonstrated that SA–AM clusters with a size of (SA)_4_(AM)_4_ would already be expected to be stable against evaporation with an evaporation rate of 1 s^–1^.^14^ Hence, based on the literature, it would be expected that SA–AM clusters larger than (SA)_6_(AM)_6_ would be stable against evaporation and would grow along the diagonal via sequential additions of SA and AM molecules.

The calculated electronic single-point contributions to the binding energies are potentially the largest source of errors in modeling the free energies of atmospheric molecular clusters.^16,17^ Previous benchmarks have focused only on smaller SA–AM clusters with *n* up to 6, which means that large errors could potentially arise for larger clusters and therefore calls for benchmarking of methods for use on larger clusters. High-level accurate single-point energy calculations are either computationally expensive or completely out of reach for large cluster sizes, but machine learning methods have emerged as an efficient tool to complement expensive quantum chemical calculations. Recently, Kubečka et al.^18^ applied the kernel ridge regression (KRR) machine learning (ML) model, with the FCHL representation^19,20^ to SA–W clusters. It was shown that the Δ-ML model^21^ trained on the binding energies of smaller (SA)_0–4_(W)_0–5_ clusters could predict the binding energies of clusters up to twice the size, with errors below 2 kcal/mol. This is an encouraging approach to obtaining accurate binding energies on larger clusters in the future.

Very little work has been performed on atmospheric clusters larger than 10–12 molecules. DePalma et al.^22^ were the first to break this limit by calculating the binding free energies of larger SA–AM clusters up to the size of (SA)_8_(AM)_8_(W)_10_. The configurational sampling was carried out using a Monte Carlo-based conformer search and the structures were optimized using a multistep optimization method using AM1 and PW91/6-31++G(d,p). The calculated binding free energies, at the PW91/6-31++G(d,p) level of theory, were found to decrease almost linearly with cluster size. In our previous study,^23^ we expanded upon this work by calculating SA–AM clusters up to the size of (SA)_20_(AM)_20_, where the sampling was carried out using ABCluster.^24,25^ For the (SA)_*n*_(AM)_*n*_ clusters, with *n* = 6–8, it was found that our sampling method resulted in cluster structures lower in free energy than the ones found by DePalma et al.^22^ when we reoptimized our cluster structures at the PW91/6-31++G(d,p) level of theory. Due to the massive size of the clusters (*n* up to 20), the level of theory for calculating the cluster structure and binding energies had to be lowered. The binding free energies of the clusters were benchmarked against high-level DLPNO-CCSD(T_0_)/aug-cc-pVTZ//ωB97X-D/6-31++G(d,p) calculations for the (SA)_*n*_(AM)_*n*_ clusters, with *n* up to 6 by Besel et al.^15^ It was found that the B97-3c//GFN1-xTB level of theory yielded binding free energy results that corresponded well with the benchmark values. Applying the B97-3c//GFN1-xTB level of theory on clusters up (SA)_20_(AM)_20_, we found that the linearly decreasing trend in the binding free energies continued up to *n* = 20. In addition, it was found that the average binding free energy per molecule leveled out around *n* = 8 to a constant value. The leveling out in the average binding free energies coincided with structural changes in the cluster, with an ammonium ion being fully “solvated” in the cluster core. Such a structure was also found in the study by Depalma et al.^22^ This could indicate that around *n* = 8, we are transitioning between the discrete cluster thermodynamics and the continuum aerosol particle thermodynamics regimes.

Hence, we build upon our previous study^23^ by investigating the growth of very large clusters. We further increase the sizes of the studied clusters by investigating the (SA)*_m_*(AM)*_n_* clusters with *n* = *m* = [21–30] and *n* = *m* ± 1, *m* ± 2 for *n* = *m* = [6–20]. The aim is to obtain insight into whether the linearly decreasing trend of the clusters continues from *n* = 20–30 and attempt to calculate the addition energies to describe the growth of the particles. Moreover, we benchmark the electronic binding energies calculated at the DFT level for the large (SA)_8_(AM)_10_, (SA)_9_(AM)_10_, (SA)_10_(AM)_10_, and (SA)_10_(AM)_11_ clusters against high-level DLPNO-CCSD(T_0_)/aug-cc-pVTZ results. This was done to find methods with which we could achieve a higher accuracy of addition free energies in order to evaluate the reliability of the configurational sampling. Finally, as a proof of concept, we also test Δ-ML approaches for modeling the binding energies of large SA–AM clusters.

## 2 Computational Details

### 2.1 Extension of the Data Set

We followed the general workflow and level of theory of our previous study^23^ for the newly generated clusters. For each cluster, we generate an initial ensemble of starting structures using ABCluster^24,25^ with the CHARMM^26^ force field in order to probe the configurational space. This was done using a population of 3000 structures running for 200 generations with a maximal structure lifetime of 4 generations for all cluster sizes. This choice of parameters was based on the study by Kubečka et al.^27^ We used ionic monomers: HSO_4_^–^ and NH_4_^+^ to enforce proton transfer as this is usually found to be the case for the lowest free energy structures of (SA)*_n_*(AM)*_n_* clusters larger than the dimer.^15,28,29^ ABCluster calculations for the clusters with more than 20 SA were carried out on the LUMI supercomputer. The 1000 lowest-energy structures were saved for further geometry optimization at the GFN1-xTB^30^ level performed in the xTB program,^31^ and the thermal corrections from GFN1-xTB were used for all free energy calculations. The decision to use GFN1-xTB instead of GFN2-xTB^32^ was motivated by our previous study,^23^ where this method was found to better describe the thermal contributions for (SA)_n_(AM)_n_ clusters. This was corroborated for the binding energies by Jensen et al.,^33^ who found it to be due to the additional *d* basis functions removed from sulfur in the transition from GFN1-xTB to GFN2-xTB.

We compared all structures by calculating root mean square deviations (RMSD) for all pairs of structures using ArbAlign.^34^ The minimum allowed RMSD for judging the uniqueness of the structures was set to 0.38 Å based on previous studies on sulfuric acid–water clusters.^35,36^

The single-point energies of the remaining clusters were calculated at the B97-3c^37^ level of theory which were run using ORCA 5.0.0 and 5.0.3.^38^ The *m* = [6–20] structures and energies were taken from our previous study.^23^ The binding free energy of the (SA)*_m_*(AM)*_n_* clusters is defined as
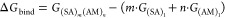
1and we calculate the binding free energy by the combination of an electronic contribution and a thermal contribution as
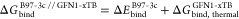
2using the electronic contribution, Δ*E*_bind_^B97-3c^, calculated at the B97-3c level of theory combined with the thermal contribution, Δ*G*_bind, thermal_^GFN1-xTB^, calculated at the GFN1-xTB level of theory.

In addition to this, we also calculated the free energy using two other methods for the single-point energy: r^2^SCAN-3c and ωB97X-D3BJ/6-311++G(3df,3pd). r^2^SCAN-3c was done for all of the generated structures. ωB97X-D3BJ/6-311++G(3df,3pd) was done for a subset of the generated (SA)*_m_*(AM)*_n_* structures. For those with less than 9 SA, all of the structures were calculated. For structures with between 9 and 14 SA, it was a subset of the structures ranging between ≈50 – 100 % of the total number of structures, and for structures with more than 15 SA, no calculations were carried out.

As cluster growth can be expected to occur through the addition of monomers and hetero dimer, we define and calculate different addition energies given by the following set of reactions
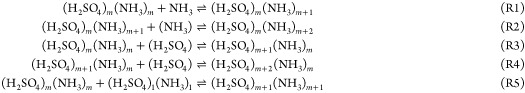
R1These reactions are denoted: Addition of the 1st AM (R1), the addition of the 2nd AM (R2), the addition of the 1st SA (R3), the addition of the 2nd SA (R4), and the addition of a SA–AM pair (R5).

### 2.2 Benchmark Calculations

The single-point energy benchmark calculations were calculated for (SA)_8_(AM)_10_, (SA)_9_(AM)_10_, (SA)_10_(AM)_10_, and (SA)_10_(AM)_11_ using ORCA 5.0.3. The structures used for the benchmark were taken from the extended data set described in Section 2.1 and Section 3.1. We used all of the generated structures for the different clusters except for (SA)_10_(AM)_11_ where the number of structures was reduced using ArbAlign with a higher cutoff of 0.68 Å due to the high computational cost of the benchmark method. Therefore, only 86 of the (SA)_10_(AM)_11_ clusters were used for the benchmark instead of the 200–300 structures for the other clusters. The (SA)_10_(AM)_12_ clusters were omitted entirely because the calculations for the benchmark method became too computationally demanding.

This benchmark was carried out using: PW91,^39^ M06-2X,^40^ and ωB97X-D3BJ^41^ with either 6-31++G(d,p)^42,43^ or 6-311++G(3df,3pd)^42,43^ basis set. We also tested the empirically corrected DFT methods B97-3c^37^ and r^2^SCAN-3c.^44^ Furthermore, we tested the wavefunction method DLPNO-CCSD(T_0_)^45,46^ with the RI-JK approximation using either aug-cc-pVTZ^47^ or aug-cc-pVDZ.^47^ The method chosen as the reference in this benchmark was DLPNO-CCSD(T_0_)/aug-cc-pVTZ.

### 2.3 Machine Learning

The machine learning models in this study were trained using the QML python toolkit.^48^ It utilizes kernel ridge regression (KRR) coupled with the FCHL19^20^ representation as was done in Kubečka et al.^18^ The KRR used in this study is characterized by the hyperparameters: σ, the kernel width, and λ, the regulariser. The values for these hyperparameters were taken from the previous study by Kubečka et al.^18^ The models used in this study are “Δ learning” models,^21^ which means that they are trained on the energy difference between the electronic binding energies at two different levels of theory, here GFN1-xTB and B97-3c
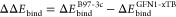
3

We trained three different models on the (SA)*_m_*(AM)*_m_* clusters. The number of equilibrium structures in the training set for different sizes can be seen in the SI. One model was trained on the equilibrium structures with *m* = [1–5], another with *m* = [1–10], and the last model was trained on the equilibrium structures with *m* = [1–5] with an additional nine out-of-equilibrium structures generated for each equilibrium structure using molecular dynamics (MD) simulations at the GFN1-xTB level. The simulations were run using ORCA 5.0.0 as the driver and interface with the XTB program. It was run using initial velocities corresponding to a temperature of 300 K and equilibrated with a 300 K thermostat every 10 fs. The simulations were run for 1000 fs in steps of 0.5 fs, with snapshots of the structures being taken every 200 steps, i.e., every 100 fs.

The models were tested on the equilibrium structures with *m* = [6–30], with the number of structures in the testing sets ranging from 122 structures to 491 structures which can be seen in the SI.

## 3 Results and Discussion

### 3.1 Binding and Addition Free Energies

This study builds upon the previous study by Engsvang and Elm^23^ where we calculated the equilibrium structures for (SA)*_m_*(AM)*_n_* clusters with *n* = *m* = [6–20]. For the same type of clusters, we have examined the larger “diagonal” clusters with *n* = *m* = [21–30] (i.e., with a 1:1 ratio of acid to base) to investigate whether the energy trends observed in our previous study would persist for even larger clusters. Likewise, we calculate the “off-diagonal” clusters with *n* = *m* ± 1, *m* ± 2 for *m* = [6–20].

The lowest values of the binding free energy for the (SA)*_m_*(AM)*_n_* clusters can be seen in the SI. We observe a nearly linearly decreasing trend showing increased stability as the diagonal clusters grow to larger sizes. This trend also holds for the off-diagonal clusters with both a higher or lower ratio of acid to base.

**Figure 1:**
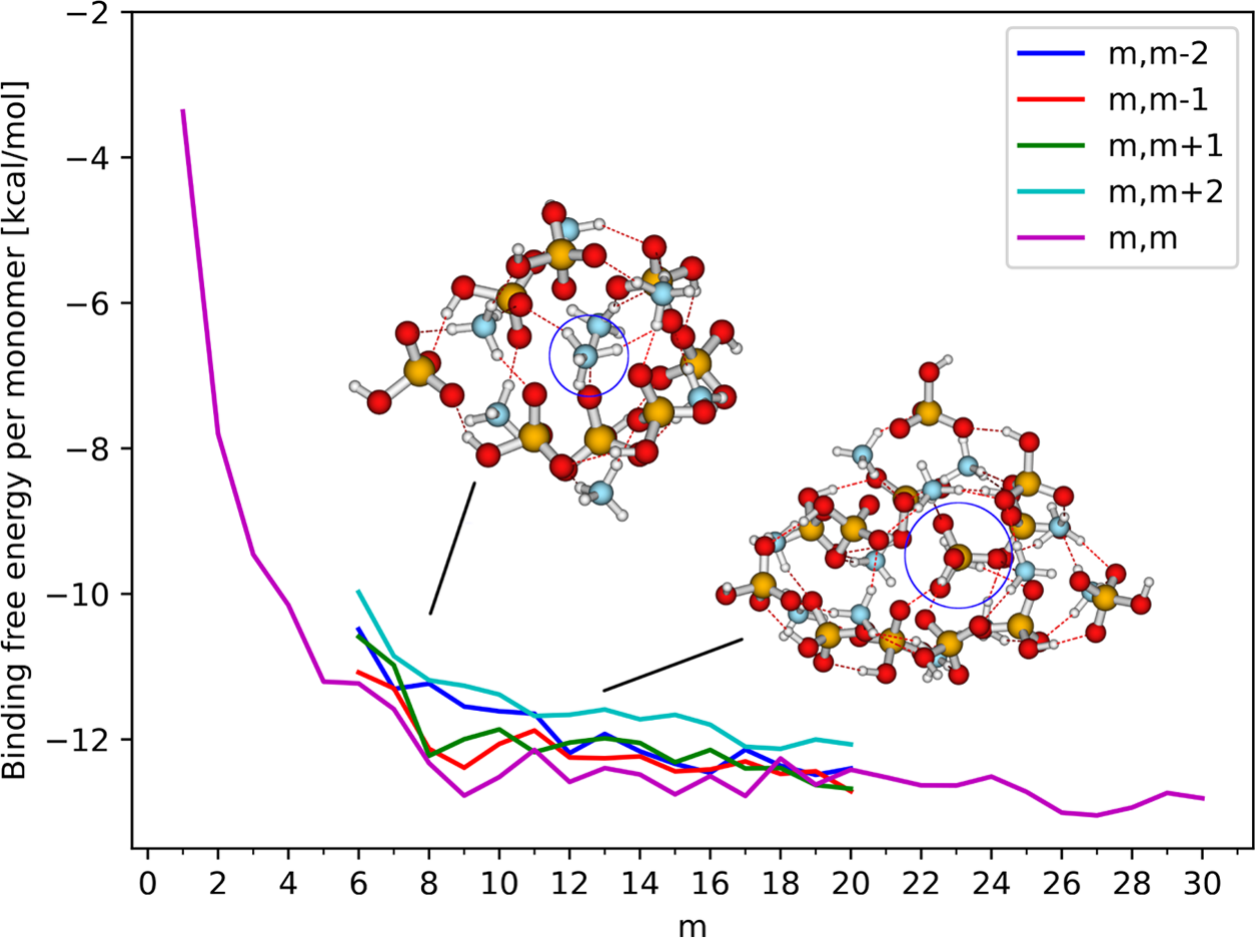
Binding free energy per monomer of the structures with the lowest value of the binding free energy of the (SA)*_m_*(AM)*_m_*, (SA)*_m_*(AM)_*m*±1_, and (SA)*_m_*(AM)_*m*±2_ clusters. The binding free energies are calculated at the B97-3c//GFN1-xTB level, at 298.15 K and 1 atm. The blue circles in the cluster structures mark the “solvated” ammonium ion for the (SA)_8_(AM)_8_ and the bisulfate ion for (SA)_13_(AM)_13_.

To obtain additional insight into the stability of the clusters, we have plotted the total binding free energy divided by the number of monomers in the cluster, i.e., the average binding energy for each monomer (seen in Figure 1).

While the binding free energy per monomer rapidly decreases from *m* = 1 to *m* = 9, it levels out for larger cluster sizes reaching a constant value of approximately −12.8 kcal/mol for the diagonal clusters and between −12.5 and −12 kcal/mol for the off-diagonal clusters. This shows that the diagonal configurations of 1:1 SA and AM should provide the most stable cluster composition for the studied cluster sizes. However, a very subtle decreasing trend can be observed for the binding free energy per monomer from −12.1 kcal/mol at *m* = 11 to −12.8 kcal/mol at *m* = 30. Furthermore, it can be observed that the off-diagonal structures with an excess of acid are more favorable than those with an excess of base.

We note that the binding free energy value is half of the value previously reported in the study by Engsvang and Elm^23^ because we report the binding energy per monomer, while the previously reported value is the binding energy per acid–base dimer.

The change in the trend is believed to be due to changes in the coordination environment inside the cluster, which was also noted in our previous study.^23^ At small cluster sizes up to (SA)_6_(AM)_6_ all of the molecules are exposed to the exterior, which was also observed by DePalma et al.^49^ For clusters larger than this we start to observe coordination of molecules in the interior. This same tendency toward coordination of the acid by ammonium was also observed for larger nitric acid–ammonia clusters by Ling et al.^50^ For the smaller clusters, this is observed to be ammonium which is marked for the (SA)_8_(AM)_8_ cluster in Figure 1, but as the cluster grows it instead becomes favorable to coordinate one or more bisulfate ions, which has been marked for the (SA)_13_(AM)_13_ cluster in Figure 1.

**Figure 2:**
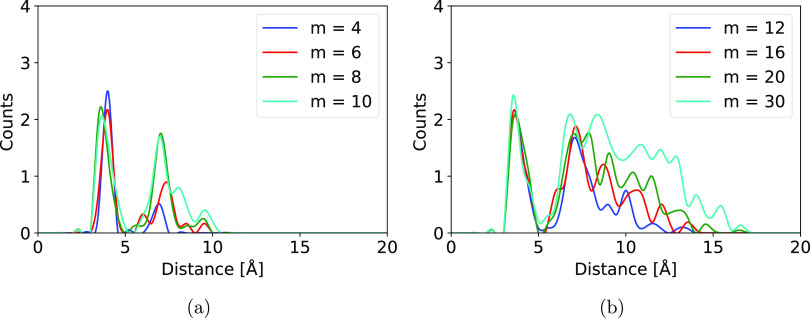
Average binned radial distribution functions (RDFs) of AM around SA in the (SA)*_m_*(AM)*_m_* clusters. A bin size of 0.5 Å and cubic interpolation between the bin points. (a) RDF of clusters with *m* = 4, 6, 8, and 10. (b) RDF of clusters with *m* = 12, 16, 20 and 30.

To quantify this change, we have calculated radial distribution functions for the clusters. These functions are the binned radial distribution of AM around each SA, where we then calculate an average over all of the SA in the clusters. The bin size was chosen to be 0.5 Å and the resulting distributions can be seen in Figure 2 where they are plotted using cubic interpolation between the bin points.

For all of the cluster sizes, a “peak” at approximately 4 Å can be seen for even the smallest clusters, which corresponds to AM immediately next to SA. However, as the cluster grows from *m* = 4 to *m* = 8, we see the development of a secondary peak at approximately 7 Å (see Figure 2a). This corresponds to AM separated from the AM–SA pair in the first peak by an intermediate layer of SA: SA–AM–SA–AM. For the clusters larger than *m* = 12, we observe AM further away leading to a less well-defined structure (see Figure 2b). In addition to these extra peaks, it can be observed that the peaks broaden as the cluster grows. The peak width correlates with the actual number of ammonia present at this distance. It does not determine whether it is SA that coordinates AM or SA that coordinates AM, but we can see that there is an increase in coordination as the cluster grows. From the visual examination, we can conclude that AM is being encapsulated within the clusters in order to coordinate a few internal SA. This is most likely due to its small size and ability to form four hydrogen bonds.

**Figure 3:**
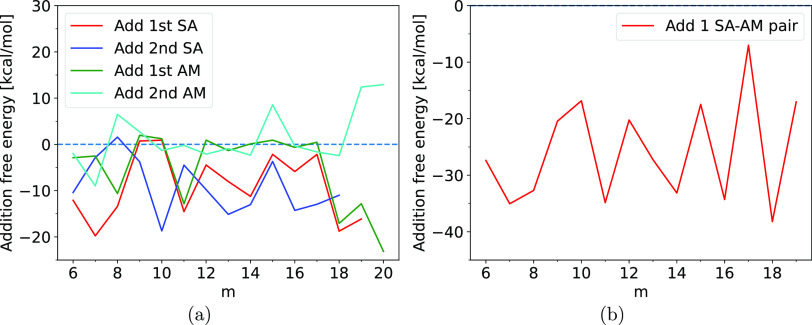
(a) Free energy difference at the B97-3c//GNF1-xTB level of theory for the growth of (SA)*_m_*(AM)*_m_* by the addition of the 1st SA, the 2nd SA, the 1st AM, and the 2nd AM. (b) Free energy difference for the addition of a SA–aM pair to (SA)*_m_*(AM)*_m_*.

The addition free energies calculated at the B97-3c//GFN1-xTB level can be seen in Figure 3, where an erratic addition trend can be observed. The calculated mean values and standard deviations of the addition energies can be seen in Table 1. Here it can be observed that the addition of sulfuric acid is generally a favorable reaction because the mean reaction free energy of the addition of the 1st and 2nd SA is found to be −9.1 ± 6.8 kcal/mol and −9.1 ± 5.7 kcal/mol, respectively. On the other hand, the addition of ammonia by itself is only an occasionally favorable growth path where the mean reaction free energy of the addition of the 1st and 2nd AM is found to be −5.2 ± 7.7 kcal/mol and 1.4 ± 5.9 kcal/mol, respectively. For all of the cluster sizes, adding the 1st sulfuric acid is more favorable than adding the 1st ammonia. This indicates that these large SA–AM clusters prefer to be more acidic than basic. While the error span might be large this illustrates that the applied methodology can still be applied to screen for potentially most stable large cluster compositions.

**Table 1:** Mean Values with Standard Deviations for the Addition to the (SA)*_m_*(AM)*_m_* of 1st SA, 2nd SA, 1st AM, 2nd AM, and a SA–AM Pair

addition of	Δ*E*_B97-3c//GFN1-xTB_ [kcal/mol]
1st SA	–9.1 ± 6.8
2nd SA	–9.1 ± 5.7
1st AM	–5.2 ± 7.7
2nd AM	1.4 ± 5.9
1 SA–AM pair	–25.8 ± 9.0

It can also be seen that the favourability of ammonia addition for *m* = 18–20 increases dramatically. However, it can be observed that the favourability of sulfuric acid addition for the same system sizes likewise increases. This indicates that the diagonal (SA)_18_(AM)_18_ and (SA)_19_(AM)_19_ structures found in the configurational search, may be comparatively unstable and that the search was not exhaustive.

As expected, adding a pair of sulfuric acid and ammonia is very favorable, with a mean value of −25.8 ± 9.0 kcal/mol. The standard deviations of these values are high due to the fluctuating behavior also seen in Figure 3.

The binding energies have previously been shown to be the largest source of error in modeling atmospheric molecular clusters.^16,17^ Hence, we suspected that the erratic behavior seen in Figure 3a and the large errors in Table 1 could potentially be due to errors in the applied B97-3c//GFN1-xTB methodology.

### 3.2 Benchmark of Binding Energies

**Figure 4:**
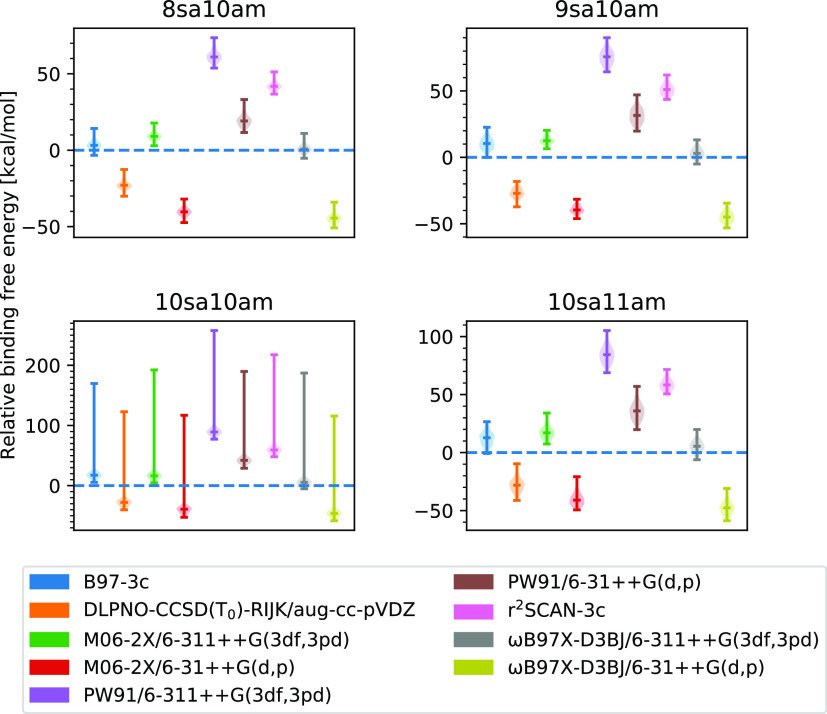
Cluster binding free energy distributions with single-point electronic energy correction calculated with different methods for different sulfuric acid and ammonia cluster configurations relative to the energy calculated at the benchmark level: DLPNO-CCSD(T_0_)/aug-cc-pVTZ//GFN1-xTB as defined in eq 4. The large outliers observed for (SA)_10_(AM)_10_ are believed to be due to these structures being previously optimized at the PM7 level of theory.

A possible reason for the erratic behavior of the addition energies is the accuracy of the calculated electronic binding energies for the applied method. Therefore, a benchmark has been carried out for our methodology. A benchmark was also carried out in the previous study by Engsvang and Elm;^23^ however, the sizes of the cluster were limited to (SA)_6_(AM)_6_ and off-diagonal clusters were not considered. Therefore, it is not necessarily an adequate benchmark for our purposes. We chose the DLPNO-CCSD(T_0_)/aug-cc-pVTZ level of theory as the benchmark method, as it has shown good agreement with higher-level coupled cluster methods.^51,52^ It should be noted that carrying out DLPNO-CCSD(T_0_)/aug-cc-pVTZ calculations on atmospherically relevant clusters consisting of 10 acid and 10 base molecules has never been done before. In addition, our benchmark set of clusters comprises hundreds of DLPNO-CCSD(T_0_)/aug-cc-pVTZ calculations on the large (SA)_8-9_(AM)_10_ and (SA)_10_(AM)_10-11_ clusters. Figure 4 presents the binding free energies calculated using different methods for all of the configurations. The relative binding free energy of conformers of the same cluster is related to the benchmark energies and defined as
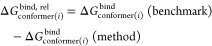
4It can be seen that the errors for the diagonal (SA)_10_(AM)_10_ structures have more extreme high-energy outliers than the other structures. As was noted in our previous study of the (SA)*_m_*(AM)*_n_* clusters with *n* = *m* = [6–20], there is an intermediate optimization step with PM7 before the xTB optimization. This optimization yielded many nonconverged optimizations, which were restarted using the nonconverged structures. This resulted in several high-energy structures for these clusters which can be observed in the data using the diagonal clusters with *n* = *m* = [6–20] taken from our previous study.

**Figure 5:**
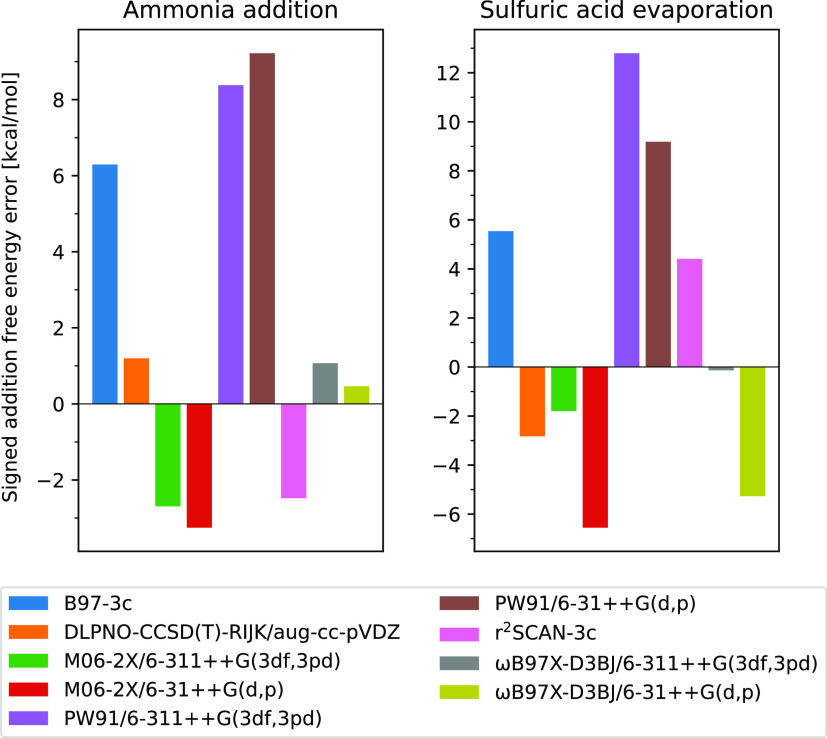
Signed addition free energy error (Δ*G*_benchmark_ – Δ*G*_method_) at the B97-3c//GFN1-xTB level for either (left) the addition of 1 ammonia to the most stable (SA)_10_(AM)_10_ cluster or (right) the removal of 1 sulfuric acid from the most stable (SA)_10_(AM)_10_ cluster.

As expected, it can be seen that the DFT methods utilizing the smaller 6-31++G(d,p) basis set or DLPNO-CCSD(T_0_) with the aug-cc-pVDZ basis set generally give binding energy results that are far off from the benchmark values. For instance, DLPNO-CCSD(T_0_)/aug-cc-pVDZ yields a mean absolute error (MAE) of 36.2 kcal/mol and ωB97X-D/6-31++G(d,p) gives an MAE of 55.5 kcal/mol. All MAEs are calculated using all of the data, which includes the extreme outliers for the diagonal structures taken from the previous study. However, due to the small number of these outliers, they have a negligible impact on the overall MAEs. While the MAEs for the methods covered in this section are large, they are not necessarily indicative of the usefulness of the method because we are primarily interested in the changes in binding energies between clusters, which we cover later in our discussion of Figure 5.

The DFT methods, utilizing a larger basis set of 6-311++G(3df,3pd), are generally observed to give results close to the benchmark except for PW91, which does not perform well regardless of the basis set size. This is most likely because PW91 is based directly on GGA with no significant modifications. Compared to ωB97X-D, it lacks e.g., dispersion and long-range corrections, which can be expected to be significant contributions to the binding energy. The ωB97X-D/6-311++G(3df,3pd) level of theory yields an MAE of 5.8 kcal/mol, similarly, M06-2X/6-311++G(3df,3pd) yields an MAE of 4.1 kcal/mol. In contrast, PW91/6-311++G(3df,3pd) yields an MAE of 68.2 kcal/mol and, interestingly, PW91/6-31++G(d,p) is better with an MAE of 22.7 kcal/mol. The performance of the empirically corrected methods is mixed since B97-3c is observed to give results close to the benchmark values with an MAE of 3.2 kcal/mol, which is consistent with the results presented in the previous study by Engsvang and Elm,^23^ while r^2^SCAN-3c is observed to be very far off the benchmark values with an MAE of 43.2 kcal/mol. This result is seemingly in contrast with the findings by Jensen et al.,^33^ where they showed this method to have far more satisfactory performance for the smaller (acid)_1-2_(base)_1-2_ systems. Decomposing the r^2^SCAN-3c method by removing the empirical corrections one by one indicates that this error is primarily caused by the geometric counterpoise correction and that the D4 dispersion corrections improve the calculations (see the SI for further details). This could indicate that when calculating binding energies, one should be aware of potential problems with the error scaling of r^2^SCAN-3c with the size of the system.

Figure 5 presents the signed free energy error, defined as: Δ*G*_benchmark_ – Δ*G*_method_, for the addition of 1 AM and the removal of 1 SA from the most stable (SA)_10_(AM)_10_ cluster. The lowest free energy of the (SA)_10_(AM)_10_, (SA)_10_(AM)_11_, and (SA)_9_(AM)_10_ clusters, at each level of theory, was chosen to calculate these energy differences.

We study this difference in binding free energy because it determines the favorability of evaporation of and condensation onto the clusters and therefore determines the growth rate. Comparing these differences with Figure 4 shows that some cancellation of errors can be expected when looking at the free energy difference between the different clusters.

Therefore, large errors for the binding free energies are less substantial as long as the method accurately describes the binding free energy differences. This is the case for nearly every method except for PW91 and B97-3c. The B97-3c method performs slightly worse for the addition energies with an error between 5.5 and 6.2 kcal/mol compared to the accuracy of the binding energy itself, where it has an error of 3.2 kcal/mol. This shows that it may not be consistent enough to be reliable for calculating the addition energies. Interestingly, r^2^SCAN-3c appears to perform slightly better than B97-3c for the addition free energies. However, the sign of the errors appears to be more inconsistent and dependent on the reaction type. To obtain highly accurate addition free energies, it is seen that either DLPNO-CCSD(T_0_)/aug-cc-pVDZ or M06-2X or ωB97X-D3BJ with the large 6-311++G(3df,3pd) basis set is needed. Especially, ωB97X-D3BJ/6-311++G(3df,3pd) appears to consistently yield errors of ∼1 kcal/mol or below. However, these levels of theory are computationally expensive and, thereby, not generally applicable to many configurations of large clusters.

**Figure 6:**
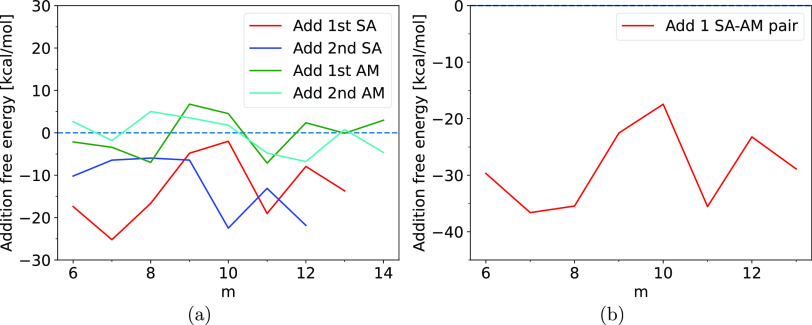
(a) Free energy difference at the ωB97X-D3BJ/6-311++G(3df,3pd)//GNF1-xTB level of theory for the growth of (SA)*_m_*(AM)*_m_* by the addition of 1st SA, 2nd SA, 1st AM, and 2nd AM. (b) Free energy difference for the addition of a sulfuric acid and ammonia pair to (SA)*_m_*(AM)*_m_*.

Based on these results, it was expected that recalculating the clusters at the r^2^SCAN-3c//GFN1-xTB level of theory might improve the addition energies. However, the r^2^SCAN-3c//GFN1-xTB results show the same behavior as in Figure 3 (see the SI). Evaluating the individual parts of r^2^SCAN-3c, i.e., r^2^SCAN, r^2^SCAN+D4, and r^2^SCAN+gCP did not improve the addition energies either (see the SI). ωB97X-D3BJ/6-311++G(3df,3pd) would be even better; however, due to the computational burden imposed by this method, we only calculated the clusters with up to 14 SA, which can be seen in Figure 6. Furthermore, for the cluster structures with 10 or more SA, not all structures were re-calculated at the higher level of theory because the calculations did not converge within a reasonable time frame of 7 days for each single-point energy calculation with 4 CPU cores.

In Table 2, it can be observed that the standard deviations of the mean values have decreased slightly for the ammonia addition, stayed nearly constant for the addition of a SA–AM pair, and finally increased slightly for the sulfuric acid addition. However, it can be seen that the mean values for the addition of SA has decreased significantly. For the first addition of SA, it decreased from −8.8 kcal/mol to −13.3 kcal/mol, and for the second addition, it decreased from −6.9 kcal/mol to −12.3 kcal/mol. Which means that the percentage deviation has decreased slightly. On the other hand, the mean values for the AM addition have increased slightly: from −2.9 kcal/mol to −0.4 kcal/mol and from −1.0 kcal/mol to −0.5 kcal/mol for the first and second addition of AM, respectively, which leads to a, percentage-wise, large increase in the uncertainty. The standard deviation of the mean for the addition of the SA–AM pair has stayed constant at 6.6 kcal/mol; however, the mean values have decreased from −26.8 kcal/mol to −28.7 kcal/mol leading to a, percentage-wise, lower error, but this change is small. Therefore, it can be concluded that the increase in the level of theory for the single-point energy did not significantly improve the results. The lack of improvement could be because the B97-3c//GFN1-xTB results exhibit some degree of error cancellation between the single-point energy and the other underlying errors.

**Table 2:** Mean Values, with Standard Deviations, for the Addition of the 1st SA, 2nd SA, 1st AM, 2nd AM, and a Pair of SA and AM to the (SA)*_m_*(AM)*_m_* Clusters^a^

addition of	Δ*E*_ωB97X-D3BJ//GFN1-xTB_ [kcal/mol]	Δ*E*_B97-3c//GFN1-xTB_ [kcal/mol]
1st SA	–13.3 ± 7.3	–8.8 ± 7.0
2nd SA	–12.3 ± 6.6	–6.9 ± 6.1
1st AM	–0.4 ± 4.7	–2.9 ± 5.0
2nd AM	–0.5 ± 3.9	–1.0 ± 3.9
1 SA–AM pair	–28.7 ± 6.6	–26.8 ± 6.6

a The values in the table are only calculated for the clusters with up to 14 SA. The ωB97X-D3BJ binding energies were calculated using the 6-311++G(3df,3pd) basis set.

Hence, the erratic behavior, seen in Figure 3 and Figure 6, could potentially be due to other sources of error than the error in the single-point energy method. One of these could be the error in the configurational sampling of the clusters.

### 3.3 Δ-ML Prediction of the Electronic Binding Energies

**Figure 7:**
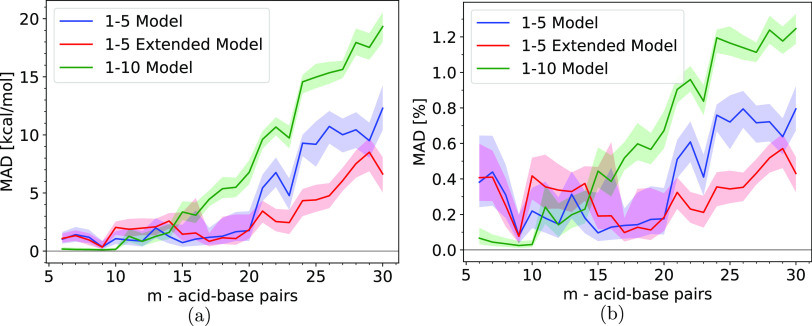
(a) Median absolute deviation (MAD) of the electronic binding energy and (b) MAD of the electronic binding energy as a percentage of the total electronic binding energy for the ML models trained on the (SA)*_m_*(AM)*_m_* equilibrium structures with *m* = [1–5], *m* = [1–10], or *m* = [1–5] where each equilibrium structure is paired with 9 additional nonequilibrium structures generated through MD using the GFN1-xTB method. The deviation from the median is illustrated by the shaded areas, where the boundary of the shaded areas is defined by the mean of the positive and negative deviations in the binding energy distribution relative to the median.

Most of the previously considered methods have generally been computationally inexpensive in order to be practically feasible. However, this was generally coupled with larger errors which contribute to making the final addition energies unreliable. Therefore, we considered the use of Δ-ML models in order to predict the binding energies of larger clusters. As a proof of concept, we trained models to predict the difference between the GFN1-xTB and B97-3c binding energies. However, the viability of the model should in principle be invariant to the chosen methods. Figure 7 shows the median absolute deviations (MAD) of the electronic binding energies and the MAD as a percentage of the binding energy for three Δ-ML models trained on equilibrium diagonal SA–AM structures of different sizes and either with or without additional nonequilibrium structures.

**Figure 8:**
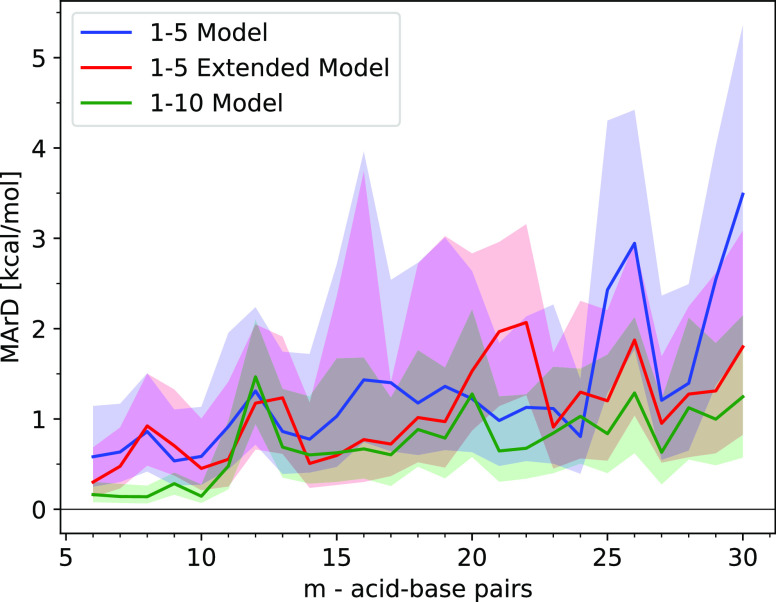
Median absolute relative deviation (MArE) of the electronic binding energy, relative to the minimum-energy structure, for the ML models trained on the (SA)*_m_*(AM)*_m_* equilibrium structures with *m* = [1–5], *m* = [1–10], or *m* = [1–5] where each equilibrium structure is paired with 9 additional nonequilibrium structures generated through MD using the GFN1-xTB method. The deviations from the median are illustrated by the shaded areas, where the boundary of the shaded areas is defined by the mean of the positive and negative deviations in the binding energy distribution relative to the median.

The Δ-ML models performed well for these types of systems, which can be seen in Figure 7a, where 3 different models are presented, and a more detailed figure showing the distribution can be seen in the SI. In Figure 7 and Figure 8, we present the MAD with the solid lines, and the shaded areas representing the deviations from the median are defined by a boundary given by the mean of the positive and negative deviations. The reason for choosing the median as a measure of the error in favor of the mean was due to the presence of a few very high error outliers. Therefore, we have included figures with box plots representing the entire distribution in the SI. Nevertheless, these high error outliers corresponded to high-energy conformations which are less relevant for our purposes. Their large error is mainly due to configurations that are not well represented by our training sets which are based on small equilibrium and near-equilibrium cluster structures. We initially trained the model on the *m* = [1–5] equilibrium structures, followed by attempts at improving the accuracy by increasing the training set size. For the initially trained model, we observed median errors of less than 3 kcal/mol for structures smaller than *m* = 20; however, we observed a steep rise in the error once the size exceeded *m* = 20 where the error quickly grows from ∼2 kcal/mol up ∼12 kcal/mol. As can be observed from Figure 7a, increasing the training set with the *m* = [6–10] equilibrium structures did not improve the results, and it actively hampered the accuracy of the model with the error quickly growing beyond 10 kcal/mol at *m* = 22 and increasing to a maximum of approximately 20 kcal/mol at *m* = 30. On the other hand, increasing the training set size by including out-of-equilibrium structures based on the *m* = [1–5] structures resulted in lower errors on average, with an error of approximately 1 to 3 kcal/mol for the small clusters with *m* ≤ 20, after *m* = 20, it rises slowly with a maximum of ∼8 kcal/mol error for *m* = 29.

The massive rise in errors for increasing cluster sizes could be because the configurational space spanned by the training set may be inadequate to describe the larger clusters. The initial training set was solely populated by equilibrium structures for the smaller clusters, which may not be a representative data set for the larger clusters. Incorporating the *m* = 6–10 clusters appears only to reinforce the existing training set resulting in a significant decrease in the transferability of the method to larger cluster sizes. However, the out-of-equilibrium structures for the smaller clusters, generated through MD, do appear to significantly expand the span of the configurational space resulting in slightly larger errors for the smaller clusters but smaller errors for the larger clusters not included in the training set. This shows that including out-of-equilibrium in the model training set leads to a more transferable model.

In Figure 7b, where the error can be seen as a percentage of the total binding energy, it can be observed, for the model with *m* = [1–5] with additional nonequilibrium structure, that while the numerical value of the median error increases significantly for larger clusters, the median percentage error lies between 0.1 % and 0.6 % showing only a slight tendency toward higher percentage error for larger clusters. From the benchmark results in the previous section, the percentage error in electronic binding energies for the ordinary methods can also be calculated. For B97-3c, this was found to be 2.6%; for ωB97X-D3BJ/6-311++G(3df,3pd), it is 2.3%; and for M06-2X/6-311++G(3df,3pd), it is 1.8%. Hence, the error associated with the Δ-ML prediction is relatively small compared to the error associated with the methods we try to predict.

The increase in the median error for the largest clusters could be due to the currently incorporated out-of-equilibrium structures not being enough to describe the extended configurational space for the largest clusters, thereby slightly decreasing the accuracy of the model. A simple solution to this problem would be to incorporate more out-of-equilibrium structures. This would increase the accuracy of the model but also increase the computational cost of the model.

These results are still promising since we have shown that we can predict the electronic binding energies with relatively small median errors up to (SA)_20_(AM)_20_ based on a model trained solely on (SA)_5_(AM)_5_ as the largest clusters. It is feasible to calculate binding energies at high levels of theory for clusters as large as (SA)_5_(AM)_5_, which means that in future studies, it could be possible to train models on DLPNO-CCSD(T_0_) binding energies or ωB97X-D3BJ with a large basis set and achieve more accurate results for the larger clusters.

Another possible application of these models can be found by looking at the median absolute relative deviations (MArD), which are the errors relative to the minimum-energy structure (see Figure 8). The MArD is a proxy for how well the model predicts the energetic ordering of the structures. The MArD for all of the different models grows very little for larger structures compared to the median absolute error seen in Figure 7a. Furthermore, it can be observed that the *m* = [1–10] model does not exhibit the same errors when predicting the relative errors, which means that while expansion of the data set decreases the accuracy of the model, it does not affect how precise the predictions are relative to each other.

This means that the models could more reliably be applied as a screening tool where they could be used to predict the energetic ordering of the configurations of a given cluster. This would be an efficient approach to identifying the lowest-energy cluster configuration. Such an approach has previously been applied by Kubečka et al. for smaller clusters with up to 4 SA and 4 base molecules.^53^

## 4 Fragmentation Methods vs Δ-ML

Obtaining the binding energies of large clusters has previously been achieved via quantum mechanical fragment-based methods.^54,55^ In order for fragmentation methods to be useful for large atmospheric cluster systems, we need some systematic way to fragment the clusters.^56^ If arbitrary cluster fragmentation is used, the results will be highly unreliable and severely erroneous conclusions could be drawn. We tested the systematic many-body-expansion (MBE) approaches presented by Hellmers and König.^57^ We tested two different schemes, a “hierarchal approach” and an “*n*-neighbor approach”. The MBE works by introducing a truncation level to include all unique dimers (MBE(2)), trimers (MBE(3)), tetramers (MBE(4)), etc. in the cluster.

**Figure 9:**
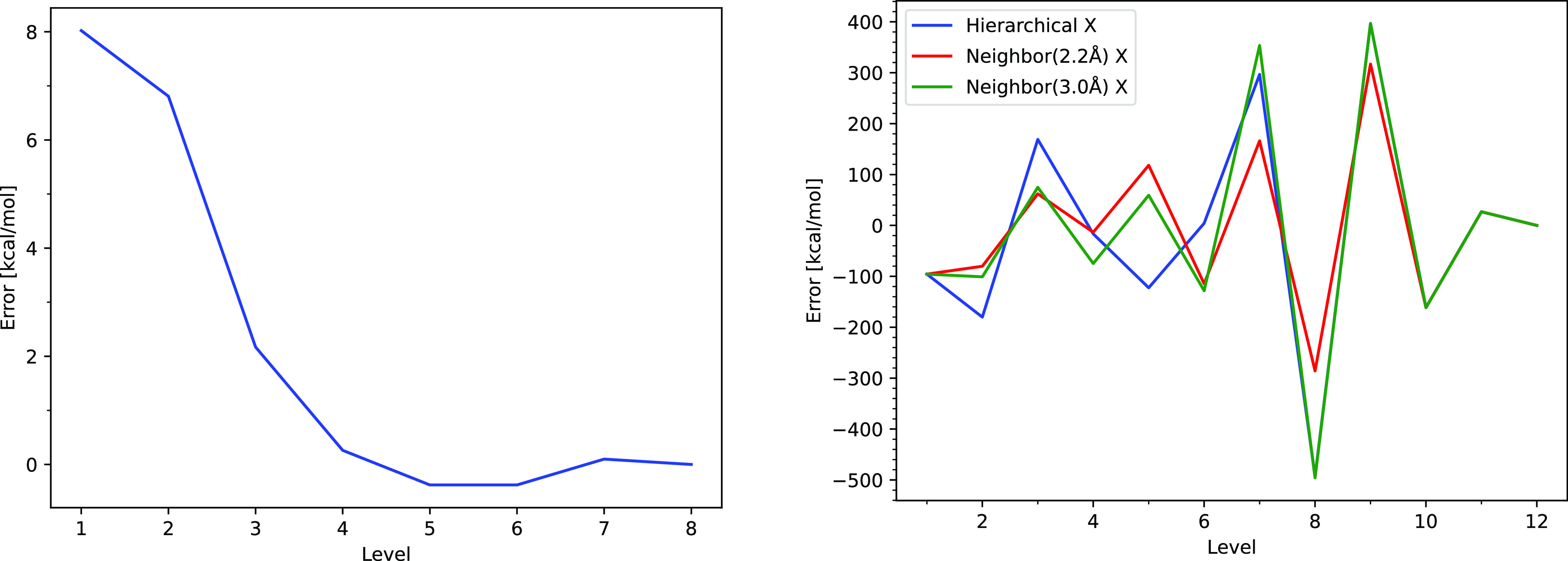
(Left) Neighbor approach as a function of truncation level with a 2.2 Å cutoff for a (H_2_O)_8_ cluster. (Right) Different fragmentation approaches tested on a (SA)_6_(AM)_6_ cluster.

Figure 9 (left) presents the test on a pure (H_2_O)_8_ cluster. It is seen that the fragmentation error smoothly converges toward zero as a function of truncation level. We did extensive testing on a (SA)_6_(AM)_6_ cluster, with various levels of theory and different parameters (Figure 9, right). Based on the systematic nature of the algorithm, one would expect that increasing the number of interacting monomers in the fragmentation scheme should lead to improved results. However, we observed very divergent behavior, where the error in the binding energy does not go down with an improved description of the fragmentation scheme. We suspect that the nonconvergent behavior for the SA–AM clusters is due to the proton transfer nature of these clusters, which yield problematic charge distributions. Hence, fragmentation methods for these systems are not straightforward to use and extremely large errors can occur if one is not careful.

## 5 Atmospheric Implications

Understanding how atmospheric molecular clusters form and grow to larger sizes is important for atmospheric chemistry. For instance, uncertainty in the relative importance of various vapors for the growth of 1.7 nm particles leads to large errors in climate models.^58^ Here we demonstrate that sulfuric acid–ammonia clusters, with a 1:1 ratio of acids to bases, grow preferentially via the addition of sulfuric acid molecules compared to ammonia molecules. This indicates that small clusters and freshly nucleated particles might be more acidic than previously expected. This finding has major implications for atmospheric chemistry, as the particle acidity has a large effect on multiphase chemical reactions such as organosulfate formation,^59−61^ oligomerization,^62,63^ viscosity,^64−66^ and phase state.^67,68^ This finding suggests that the enhanced stability found for small clusters (up to 10 molecules) with a 1:1 acid-to-base ratio, might not necessarily be valid for larger cluster systems (10–60 molecules). Hence, SA–AM clusters with a higher SA content should be further studied.

We find that the average binding free energies level out already after 10–15 acid–base pairs, implying that clusters with less than 30 molecules might actually behave as bulk particles. In addition, this also means that it is most likely not necessary to extend the cluster size beyond 15 acid–base pairs in future studies. This will make it much more efficient to screen multiple large cluster compositions and evaluate which compositions lead to stable particles. Understanding at what size and compositions clusters are stable and begin to behave as particles is important in accurately describing the cluster growth and dynamics in aerosol models.

## 6 Conclusions

We have studied the binding free energies of the (SA)*_m_*(AM)*_n_* clusters with *m* = *n* = [1–30], and clusters with *n* = *m* ± 1, *m* ± 2 for *m* = [6–20], at the B97-3c//GFN1-xTB level of theory. We find that the clusters grow in stability with regard to the total binding free energy as the cluster size is increased. Additionally, the average binding free energy decreases for small clusters and levels out at a value of around −12.8 kcal/mol for larger clusters. This is the case for both the clusters with a 1:1 ratio of SA and AM but also for the clusters with ±1 or ±2 AM molecules. The diagonal clusters with 1:1 ratios are the most stable, but for the off-diagonal clusters, the clusters with an excess of SA are preferable to clusters with an excess of AM within the studied size regime. We show that the addition free energies at the B97-3c//GFN1-xTB level of theory exhibit an erratic behavior that persists at higher levels of theory, which implies that other potential sources of error are present. We hypothesize that this could be due to insufficient sampling of the configurational space which should be explored further in the future. More exhaustive configurational sampling could be performed by running several ABCluster simulations in parallel, which would increase the chance of finding the global energy minimum.

In our benchmark, we show that the B97-3c//GFN1-xTB level of theory may yield an accuracy appropriate for qualitatively describing the binding free energy, but it should not be relied upon for the calculation of addition free energies. For this, more accurate methods should be considered, such as ωB97X-D3BJ with a large basis set. However, this and other more expensive methods would require a significant investment of computational resources.

Therefore, as a proof of concept, we tested the applicability of different Δ-ML models for predicting binding energies. We find that ML models trained on a combination of equilibrium and nonequilibrium (SA)_*n*_(AM)_*n*_ clusters with *n* = [1–5] can be used to extrapolate to very large clusters with *n* up to 20 with a consistent percentage error of 0.6 % or less. Furthermore, the absolute relative error scales significantly better with cluster size, which means that the energetic ordering can be predicted much better than the binding energy itself. A model trained on an accurate, but expensive, method such as ωB97X-D3BJ/6-311++G(3df,3pd) or a coupled cluster method, would be a cheap approach to obtain accurate binding energies. We find that it is easier to expand the training set for the machine learning model when only the correct energetic ordering is needed, which suggests the use of ML as a screening step in configurational sampling in order to select the lowest-energy structures, which should be evaluated with higher-level calculations.

## Data Availability

These are made available in the atmospheric cluster database (ACDB): https://github.com/elmjonas/ACDB

## Supporting Information Available

Geometries of all discussed structures optimized at the GFN1-xTB level of theory; log files for the B97-3c, r^2^SCAN-3c, and ωB97X-D3BJ/6-311++(3df,3pd) calculations (PDF)
