# Supplementary material for: Toward Modeling the Growth of Large Atmospheric Sulfuric Acid–Ammonia Clusters
Source: ACS Omega. 2023 Sep 14;8(38):34597–609. doi: 10.1021/acsomega.3c03521 (PMC10536041; doi:10.1021/acsomega.3c03521)
Supplement: Supplementary file 1 — ao3c03521_si_001.pdf [file ao3c03521_si_001.pdf]

Supporting Information for: Toward Modeling the Growth of Large Atmospheric Sulfuric Acid–Ammonia Clusters

Morten Engsvang,[†] Jakub Kubečka,[†] and Jonas Elm[*,‡]

†Department of Chemistry, Aarhus University, Langelandsgade 140, 8000 Aarhus C, Denmark

‡Department of Chemistry, iClimate, Aarhus University, Langelandsgade 140, 8000 Aarhus C, Denmark

E-mail: jelm@chem.au.dk

Phone: +45 28938085

S1 Extended Data set

Figure S1 plots the lowest value found of the binding free energy for each of the studied clusters, where a linearly decreasing trend can be observed for both the diagonal and off-diagonal clusters.

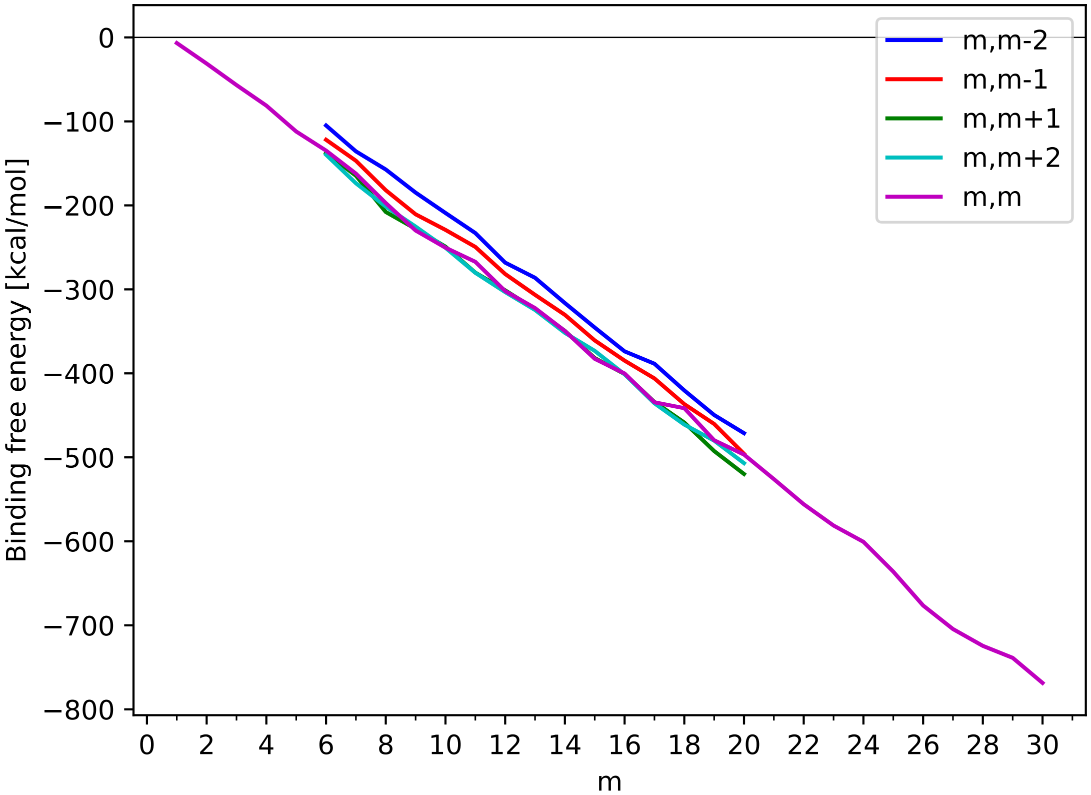

Figure S1: The lowest values of total binding free energy of the $(SA)_m(AM)_m$, $(SA)_m(AM)_{m\pm1}$ and $(SA)_m(AM)_{m\pm2}$ clusters at the B97-3c//GFN1-xTB level of theory.

S2 Benchmark

Due to the expectation of both better and more consistent performance of r^{2}SCAN-3c for the addition energies compared to B97-3c we decided to recompute the free energies at the r^{2}SCAN-3c//GFN1-xTB level of theory in order to reduce the error in the calculated addition energies arising from the choice of method. This was done in ORCA 5.0.3. The results can be seen in Figure S2 where we have also added the addition energies at the B97-3c//GFN1-xTB

level for comparison.

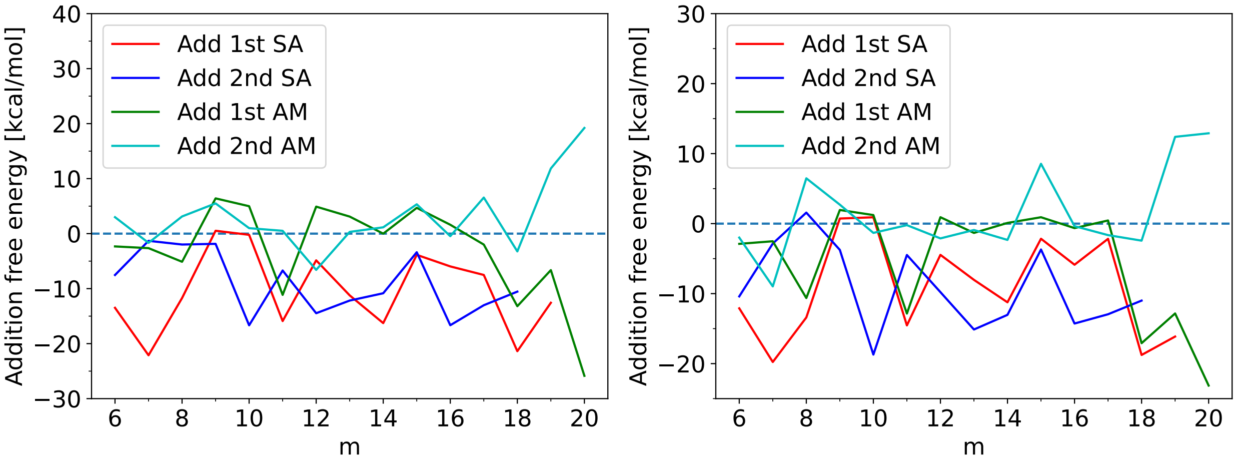

(a) (b)

Figure S2: (a) Free energy difference at the r^{2}SCAN-3c//GFN1-xTB level of theory for the addition to the $(SA)_m(AM)_m$ of 1st SA, 2nd SA, 1st AM, and 2nd AM. (b) Free energy difference at the B97-3c//GFN1-xTB level of theory for the addition to the $(SA)_m(AM)_m$ of 1st SA, 2nd SA, 1st AM, and 2nd AM.

Table S1 presents the mean values for the addition energies. The values calculated at the B97-3c//GFN1-xTB level of theory have been added for comparison.

Table S1: Mean values with standard deviations for the addition to the $(SA)_m(AM)_m$ of 1st SA, 2nd SA, 1st AM, 2nd AM, and a pair of sulphuric acid and ammonia at both the r^{2}SCAN-3c//GFN1-xTB and B97-3c//GFN1-xTB level of theory.

Addition	$\Delta E_{r^2SCAN-3c//GFN1-xTB}$ [kcal/mol]	$\Delta E_{B97-3c//GFN1-xTB}$ [kcal/mol]
1st SA	-10.5 ± 6.9	-9.1 ± 6.8
2nd SA	-9.0 ± 5.4	-9.1 ± 5.7
1st AM	-2.9 ± 8.4	-5.2 ± 7.7
2nd AM	3.0 ± 6.1	1.4 ± 5.9
1 SA–AM pair	-21.5 ± 9.2	-25.8 ± 9.0

From Figure S2 and Table S1, it can be seen that the recalculation at the r^{2}SCAN-3c//GFN1-xTB did not meaningfully change the observed erratic trend in contradiction with the indications from the benchmark.To further understand this error in r^{2}SCAN-3c method, we decomposing by removing the empirical corrections one by one. Figure S3 shows the er-

rors in the binding free energies for r^{2}SCAN-3c, r^{2}SCAN+gCP, r^{2}SCAN+D4 dispersion and r^{2}SCAN.

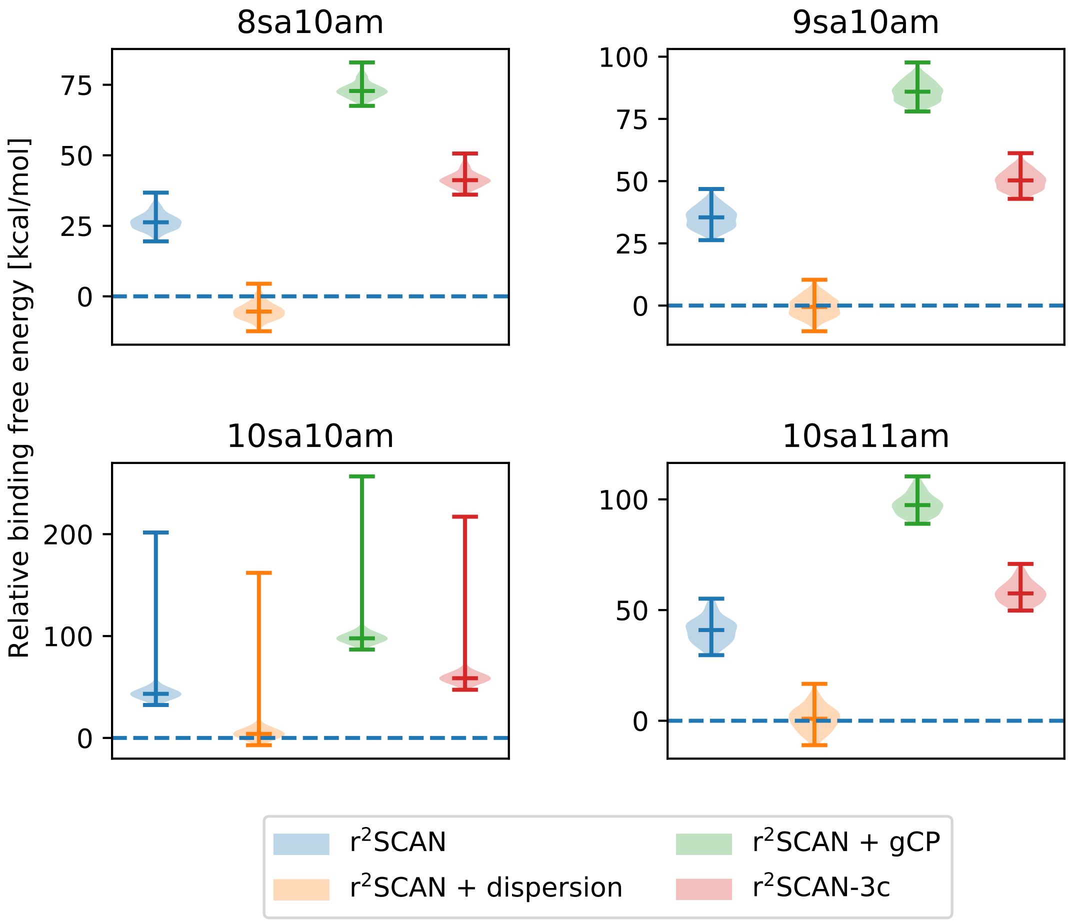

Figure S3: Errors in the binding free energy of different r^{2}SCAN variants.

By decomposing the r^{2}SCAN-3c method, we can trace that the error in the binding free energy is primarily caused by the geometric counterpoise correction (gCP). The D4 dispersion in all cases improve the calculations of the binding energies. However, this does not necessarily imply that r^{2}SCAN-D4 is unequivocally better than r^{2}SCAN-3c. Figure S4 presents the addition free energies using r^{2}SCAN-3c, r^{2}SCAN+gCP, r^{2}SCAN+D4 dispersion and r^{2}SCAN.

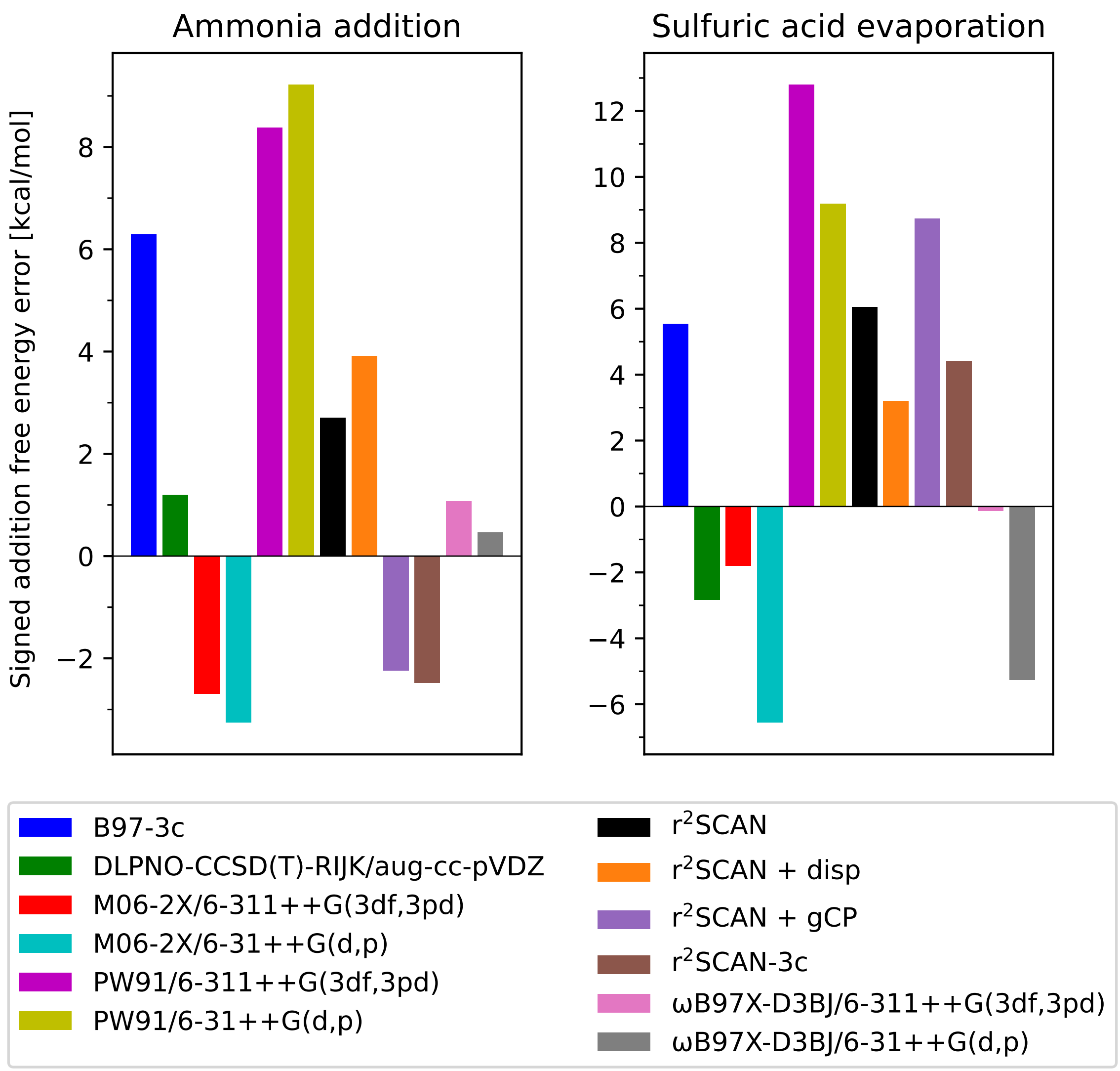

Figure S4: Errors in the binding free energy of different r^{2}SCAN variants.

It is seen that r^{2}SCAN-3c is giving the most reliable addition energies. This is most likely caused by error cancellation of the empirical gCP term.

S3 Machine-Learning

Table S2 shows the total amount of equilibrium structures in the training set for each cluster size. Table S3 shows the number of structures included in the test sets on which our ML models were evaluated.

Table S2: Number of equilibrium structures of $(SA)_m(AM)_m$ in the training set for the ML models

m	1	2	3	4	5	6	7	8	9	10
# of clusters	6	33	73	244	414	339	426	489	206	308

Table S3: Number of structures in the testing sets for the $(SA)_m(AM)_m$ clusters with m = [6–30]

m	# of structures
6	341
7	428
8	491
9	208
10	310
11	223
12	289
13	225
14	405
15	299
16	159
17	294
18	367
19	177
20	274
21	344
22	320
23	404
24	122
25	162
26	362
27	229
28	333
29	477
30	474

Figure S5 and Figure S6 plots the absolute errors for all the structures of each cluster given in kcal/mol and % of the electronic binding energy respectively. Here the entire distribution with the extreme outliers are plotted.

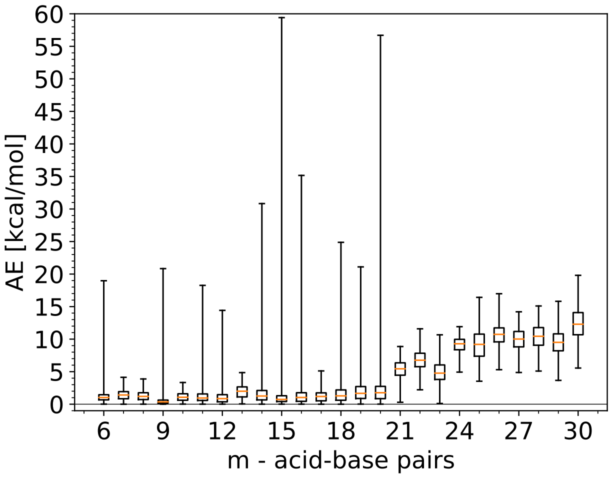

(a)

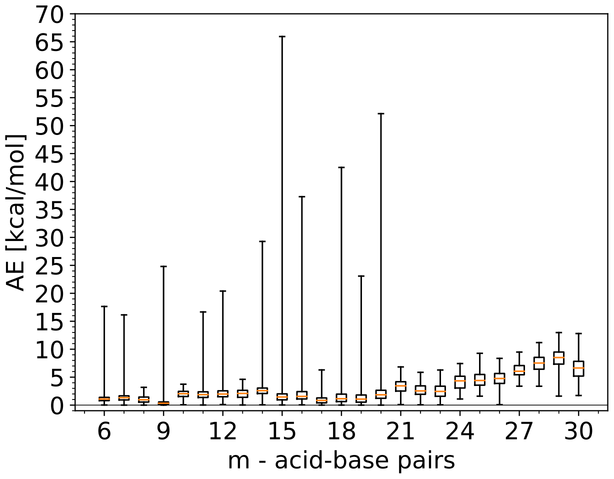

(b)

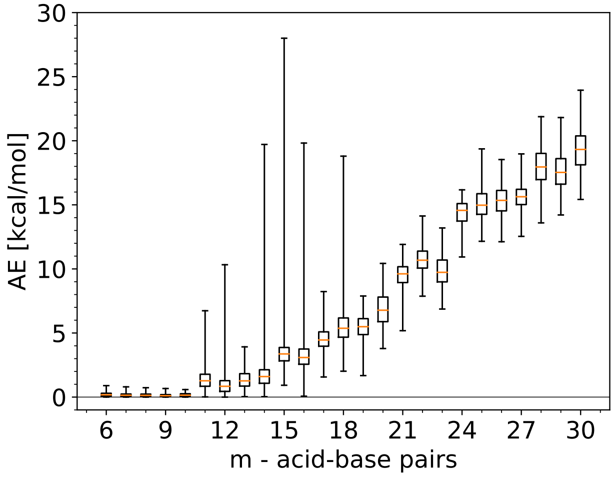

(c)

Figure S5: Absolute error (AE) in the electronic binding energy for the ML models trained on the $(SA)_m(AM)_m$ equilibrium structures with either (a) m = [1–5], (b) m = [1–5] where each equilibrium structure is paired with 9 additional non-equilibrium structures generated through MD using the GFN1-xTB method, or (c) m = [1–10]. The orange lines in the boxplots denote the mean value and the whiskers extends to the largest outliers.

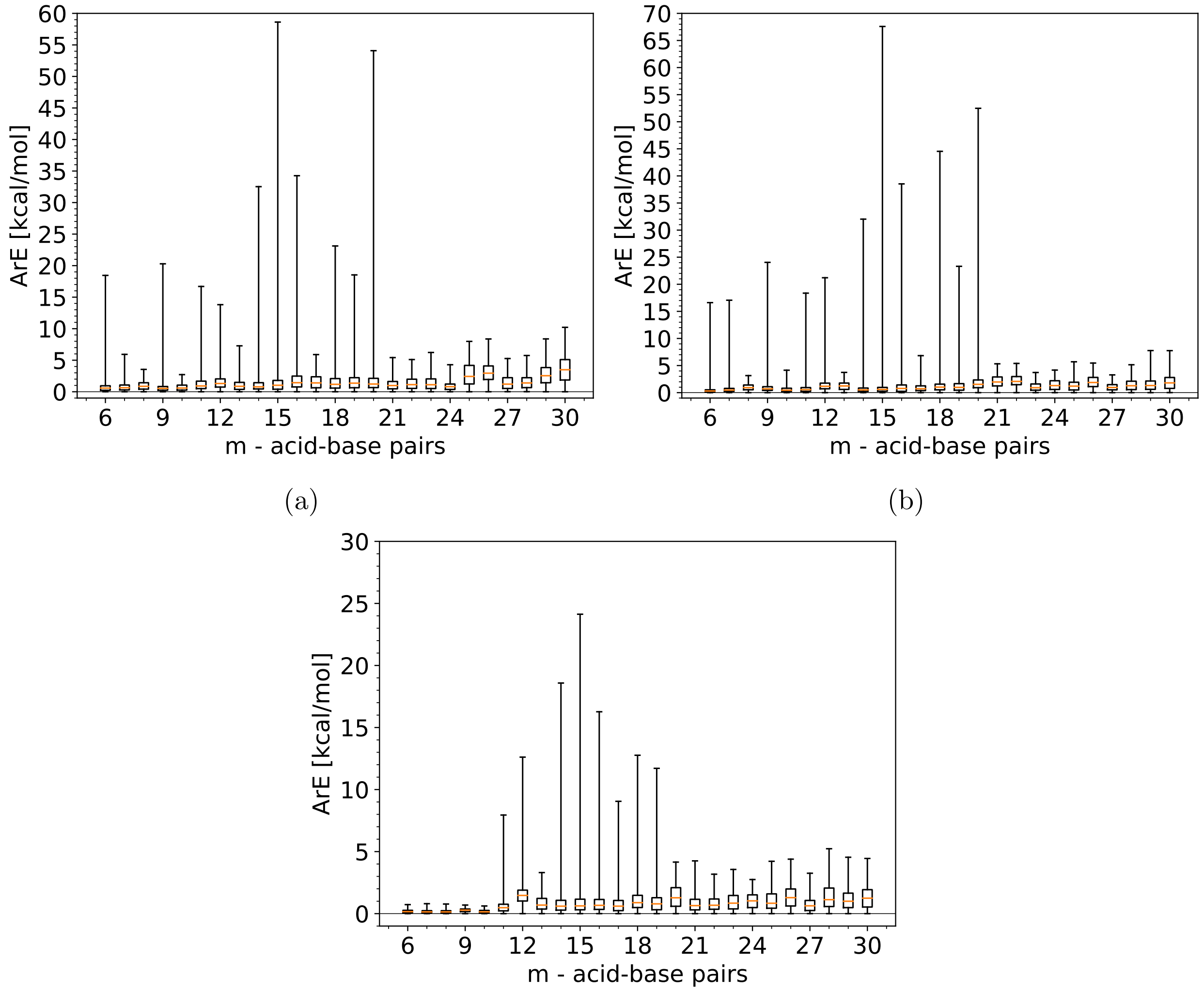

(a) (b) (c)

Figure S6: Absolute relative error (ArE) in the electronic binding energy, relative to the minimum energy structure, for the ML models trained on the $(SA)_m(AM)_m$ equilibrium structures with either (a) m = [1–5], (b) m = [1–5] where each equilibrium structure is paired with 9 additional non-equilibrium structures generated through MD using the GFN1-xTB method, or (c) m = [1–10]. The orange lines in the boxplots denote the mean value and the whiskers extends to the largest outliers.
